# Effects of Osseointegration by Bone Morphogenetic Protein-2 on Titanium Implants In Vitro and In Vivo

**DOI:** 10.1155/2016/3837679

**Published:** 2016-02-08

**Authors:** Fu-Yuan Teng, Wen-Cheng Chen, Yin-Lai Wang, Chun-Cheng Hung, Chun-Chieh Tseng

**Affiliations:** ^1^Department of Dentistry, Kaohsiung Armed Forces General Hospital, Kaohsiung, Taiwan; ^2^School of Dentistry, College of Dental Medicine, Kaohsiung Medical University, Department of Prosthodontics, Kaohsiung Medical University Hospital, No. 100 Tzyou 1st Road, Kaohsiung 80708, Taiwan; ^3^Advanced Medical Devices and Composites Laboratory, Department of Fiber and Composite Materials, College of Engineering, Feng Chia University, Taichung, Taiwan; ^4^Metal Industries Research & Development Centre, 3F, No. 88 Luke 5th Road, Luzhu District, Kaohsiung 82151, Taiwan

## Abstract

This study designed a biomimetic implant for reducing healing time and achieving early osseointegration to create an active surface. Bone morphogenetic protein-2 (BMP-2) is a strong regulator protein in osteogenic pathways. Due to hardly maintaining BMP-2 biological function and specificity, BMP-2 efficient delivery on implant surfaces is the main challenge for the clinic application. In this study, a novel method for synthesizing functionalized silane film for superior modification with BMP-2 on titanium surfaces is proposed. Three groups were compared with and without BMP-2 on modified titanium surfaces in vitro and in vivo: mechanical grinding; electrochemical modification through potentiostatic anodization (ECH); and sandblasting, alkali heating, and etching (SMART). Cell tests indicated that the ECH and SMART groups with BMP-2 markedly promoted D1 cell activity and differentiation compared with the groups without BMP-2. Moreover, the SMART group with a BMP-2 surface markedly promoted early alkaline phosphatase expression in the D1 cells compared with the other surface groups. Compared with these groups in vivo, SMART silaning with BMP-2 showed superior bone quality and created contact areas between implant and surrounding bones. The SMART group with BMP-2 could promote cell mineralization in vitro and osseointegration in vivo, indicating potential clinical use.

## 1. Introduction 

Dental implants are critical for the functional reconstruction of mouth occlusions and teeth. Titanium (Ti) is an ideal biomaterial for dental implants because of its outstanding mechanical properties, effective corrosion resistance, and exceptional biocompatibility. However, patients with osteopenia or osteoporosis, including those with diabetes mellitus and elderly patients, have poor osseous healing because of poor bone quality and quantity. These patients have less bone to apply an implant and require a longer waiting time for osseointegration after the implant is applied [1, 2]. Physical or chemical surface modification in dental implants can enhance osseointegration and reduce healing time, thereby improving patients' total recovery [3, 4].

Surface modification applied on Ti through various methods for creating unique microstructures improves biological responses and surface energy [5]. Generally, the main purpose of physically and chemically modifying surfaces is to promote implant osseointegration. In our previous study, we investigated the interaction of progenitor bone cells among several modification surfaces, all of which apply in commercial methods [6]. We found two modification surfaces, which were potentiostatic anodization (ECH) in sulfate electrolytes through constant electric current supply and sandblasting, alkali heating, and etching (SMART), in which the results reported that groups generate better potentials of cell mineralization in vitro.

Recently, ideal biomimetic surfaces have been designed to bond more biological factors and actively initiate a bone healing process. The recombinant human bone morphogenetic protein-2 (rhBMP-2), which is a transforming growth factor, has been proven to initiate and regulate osteoblast differentiation [7]. However, some studies have reported finding no critical osteoconductivity effect of BMP delivery on surfaces [8, 9]. Various methods have been applied to bonded implant surfaces for becoming biological response surfaces [8–15]. One delivery technique that we used successfully in a previous experiment was to bond the modified Ti active surfaces with the adhesion receptors in biofunctional proteins [13]. The biofunctional surfaces enhanced ALP expression of osteoprogenitor cells after 1 to 7 d of culture, after which we used acid-etched Ti surfaces that were further treated with silane coupling agents, which could covalently bond with Arg-Gly-Asp peptides on the surfaces.

The present study aimed to investigate two modification surfaces (ECH and SMART) that were further treated with silane coupling agents and then bonded with the highly bioactive protein, BMP-2. Surface wettability is commonly used in assessing the hydrophilic ability of a surface, and the contact angle of the water shows the wettability of a surface. The surface properties and characteristics were measured and evaluated by performing roughness and wettability measurements. A 3-(4,5-dimethylthiazol-2-yl)-2,5-diphenyltetrazolium bromide (MTS) assay was used to measure cell proliferation. Osteogenesis and mineralization can be detected early through alkaline phosphatase (ALP). Therefore, the cell activity and expression ability of the ALP of progenitor bone cells (D1 cells) were compared among various Ti surfaces. Animal studies were further conducted to evaluate the early new bone formation resulting from bone-to-implant contact ratios through micro-CT and histomorphometric analyses in the sandblasted and acid-etched (SLA), ECH with BMP-2, and SMART with BMP-2 groups.

## 2. Materials and Methods

### 2.1. Substrate Surface Modifications

Pure Ti samples 14.8 mm in diameter and 2 mm-thick circular substrates were used as the substrate materials for surface modification. Prior to the coating, the samples were polished with abrasive paper (grit size: 2000#), degreased with acetone, and rinsed with distilled water. The samples comprising the control group were labeled as machined surface (M). Additional modifications involved two Ti surfaces, ECH and SMART. For ECH treatment, the Ti substrate sample was used as an anode, whereas the wall of the stainless steel container was used as a cathode. An aqueous electrolyte was prepared from a solution of 0.1 M sulfate. ECH processes were executed under working voltages of 100 V. After ECH treatment of 10 min, the coated sample was removed from the electrolyte, rinsed thoroughly with distilled water, and dried at room temperature. The SMART treatment first involved using sandblasting and acid etching processes. The Ti substrate was sandblasted for 30 s using an air compressor with 3 kg/cm^2^ of Al_2_O_3_ powder (with a mean diameter of 200 *μ*m), which was followed by acid treatment in hydrochloric acid (HCL) (37%, Panreac, Barcelona, Spain), concentrated sulfuric acid (H_2_SO_4_) (95% to 98%, Panreac, Barcelona, Spain), and deionized water at 1 : 1 : 100. Simultaneously, ultrasonic acid etching was performed at 100°C for 30 min. The disk was etched in NaOH (0.5 M) for 1 h and cleaned with deionized water, dried, and immersed for 30 min in HCL (0.1 M) for etching. Finally, the samples were cleaned and dried at 100°C for 1 h.

### 2.2. Biomimetic Modification

After we performed the surface treatment processes, the samples were immersed in a 1 : 1 mixture of H_2_SO_4_ and 30% hydrogen peroxide for 2 h, washed in distilled water three times, and air dried [16]. Substrates for silanization were placed in acetone containing 10% 3-mercaptopropyltrimethoxysilane for 2 h at 25°C, rinsed in acetone, and dried overnight at 37°C. Substrates were placed in 1% glutaraldehyde in phosphate buffered saline in a vacuum oven. The BMP used in this study was rhBMP-2 provided by Genetics Institute, Cambridge, USA. The treated substrates were then dipped vertically into concentrations of BMP-2 (7.5 ng/mL).

### 2.3. Substrate Surface Analysis

A field emission scanning electron microscopy (Hitachi S-3000N, Hitachi, Tokyo, Japan) was employed to examine the substrate surface structures, cell morphology after cell attachment, and repopulation. Contact angle (CAM-100, Creating Nano Technologies, Inc., Taiwan) measurement was used to demonstrate the surface wettability of the substrates. The samples were tested in triplicate times (*n* = 3). The measurement of average surface roughness was used a roughness tester (SJ-301 Mitutoyo, Ltd., Japan).

### 2.4. Cell Culture

D1 cells (pluripotent mesenchymal cells, American Type Culture Collection, Manassas, VA, USA), cloned from BALB/c mouse bone marrow cells, were maintained in a bone medium (Dulbecco's Modified Eagle's Medium, Invitrogen, Carlsbad, CA, USA) containing 10% fetal bovine serum and 0.1% sodium ascorbate in a humidified atmosphere of 5% carbon dioxide at 37°C.

### 2.5. The 3-(4,5-Dimethylthiazol-2-yl)-2,5-diphenyltetrazolium Bromide Assay

D1 cells were seeded on the substrates with and without BMP-2 modification into a 24-well plate at a density of 100,000 cells/well with culture medium. The cells were harvested on Days 1, 4, 7, 10, 14, and 21 to detect the cell proliferation by using MTS tetrazolium (Cell Titer96 Aqueous, Promega, Madison, WI). In brief, 3 h before each of the desired time points, 10 *µ*L of the MTS reagent was added into each well, and cells were incubated at 37 *µ*C for 3 h. The absorbance was detected at 490 nm with a microplate autoreader (Dynex Technologies, Billingshurst, UK). The entire experiment was repeated three times.

### 2.6. Alkaline Phosphatase Activity

D1 cells were seeded on the Titania films with and without microarc oxidation modification into a 24-well plate at a density of 100,000 cells/well with culture medium. The cells were harvested on Days 4, 7, 10, 14, and 21. A Sigma-Aldrich Alkaline Phosphatase kit (number 85, Sigma, USA) was used to detect and stain ALP activity after simvastatin treatments. To prepare the alkaline-dye solution, 2 mL of Naphthol AS-MX phosphate alkaline was dissolved in a diazonium salt solution, which is a fast violet B capsule dissolved in 48 mL of distilled water. Cells were fixed with 10% formalin-saline at room temperature for 10 min and then the substrates were washed once with deionized distilled water (dd H_2_O) and an alkaline-dye mixture was added to each well in the 48-well plate and incubated for 15–30 min. The staining solution was removed, and the wells were washed with distilled water. The fixed and stained plates were then air-dried at room temperature, and the ALP positive stained cells were photographed using a lighter microscopy (Nikon, Japan).

### 2.7. Animal Experiments

Two female domestic pigs aged 4 mo and weighing 20 kg were used for this study. All procedures were performed in accordance with the specifications in the Guidelines for Animal Experiments of Taichung Veterans General Hospital. The pigs were starved for 1 d before surgery. All surgical procedures were conducted under Zoletil [5 mg/kg, intramuscular (i.m.)] injection and then inhalational anesthesia with isoflurane until surgery was completed. After local anesthesia by oxytetracycline (7–11 mg/kg), a sagittal incision was made on the femur, and soft tissue and periosteum were mobilized. Implant holes were created on the femur follows. First a 2 mm round bur was used to wear the cortical bone, and a 2 mm twist drill was inserted vertically 12 mm into the femur. Next, a 3 mm pilot drill was used to create a hole. Finally, five implants were placed into the femur. The condition was as follows: SLA, ECH, ECH-BMP-2, SMART, and SMART-BMP-2 (*n* = 2). After implant placement, the periosteum and skin were sutured with 4-O silk in two layers.

### 2.8. Sample Collection and Preparation of Specimens

The animals were euthanized after 14 days. The animals were initially sedated with Zoletil (5 mg/kg, i.m.) and euthanized with an injection of KCL (2 mmol/kg body weight) into the heart. After cardiac arrest, we harvested the animals' femur. These femurs were fixed by 4% paraformaldehyde. The specimens were dehydrated in increasing concentrations of alcohol at room temperature. In preparation for histological analysis, the samples were embedded in resin. Embedded bone samples were cut into thin sections 180 *µ*m thick by using a precision saw and grinding machine (CL-40, Taiwan Nakazawa), and the bone-implant sample was reduced to 30 *µ*m by using a grinder machine (FRS-C, Top Tech).

### 2.9. Microstructure and Mineral Content

The six bone-implant specimens (*n* = 6) of each group were examined using scanning electron microscopy with energy dispersive X-ray spectroscopy (SEM-EDX) on Day 14. An EDX probe coupled to a scanning electron microscope (Philips FEG XL 30, Oxford Instruments, Inc., Concord, MA, USA) at an accelerating voltage of 25 kV and a 100 *µ*A illuminating current and 100 s counting time was used. In each specimen, analyses of calcium (Ca) and phosphorus (P) content of enamel (%) were conducted.

### 2.10. Micro-CT

Micro-CT (Skyscan 1076, Bruker, Belgium) was employed to analyze bone repair, which was calculated using image software at the desired time point. The established scan conditions were an aluminum filter of 0.5 mm, 35 *µ*m scanning resolution, an X-ray voltage of 50 KV, an X-ray current of 200 mA, and an exposure time of 600 m-s. The bone healing was evaluated using analysis total volume, bone volume, and the percent bone volume (BV/TV).

## 3. Results

### 3.1. Contact Angle and Surface Morphology of Ti Implants

**Figure 1:**
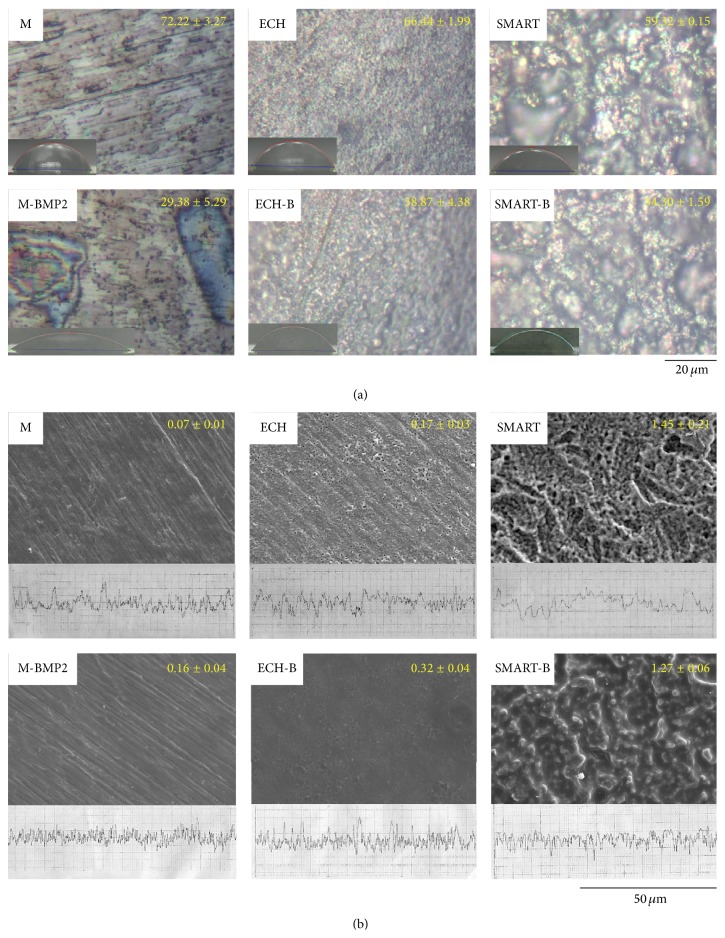
Contact angle and surface morphology of Ti implants. Surface conditions and contact angle (mean ± standard deviation; unit: degree) under a light microscope of Ti implants with or without conjugated BMP-2 (scale bar, 20 *μ*m) (a). Surface morphologies under SEM and average roughness (mean ± standard deviation; unit: *μ*m) of Ti implants with or without conjugated BMP-2 (scale bar, 50 *μ*m) (b) (ECH-B: ECH-BMP-2; SMART-B: SMART-BMP-2).

To evaluate the properties of an implant surface, wettability measurement is a typical strategy for assessing the hydrophilicity of material surfaces. The characteristics of sample surfaces are displayed in Figure 1(a). The contact angles of M, ECH, and SMART had contact angles greater than 72.22 ± 3.27°, 66.44 ± 1.99°, and 59.32 ± 0.15°, respectively. The substrate of SMART treatment had the narrowest contact angle, and the M group had the widest. The results of a previous study showed a similar trend [6]. After bonding with BMP-2, the surface-modified M-B, ECH-B, and SMART-B substrates were hydrophilic because their contact angles were 29.38 ± 5.29°, 58.87 ± 4.38°, and 54.30 ± 1.59°. The surface roughness of the M, ECH, and SMART substrates was 0.11 ± 0.01, 0.17 ± 0.02, and 1.35 ± 0.06 *μ*m. The surface roughness of the substrate samples, in which SMART had the greatest surface roughness value of above 1.35 *μ*m, is shown in Figure 1(b). After coating BMP-2, the roughness of the M-B and ECH-B increased to 0.16 ± 0.04 and 0.32 ± 0.04 *μ*m. However, the roughness of SMART-B decreased to 1.72 ± 0.06 *μ*m.

### 3.2. Cell Morphology on Various Ti Implants

**Figure 2:**
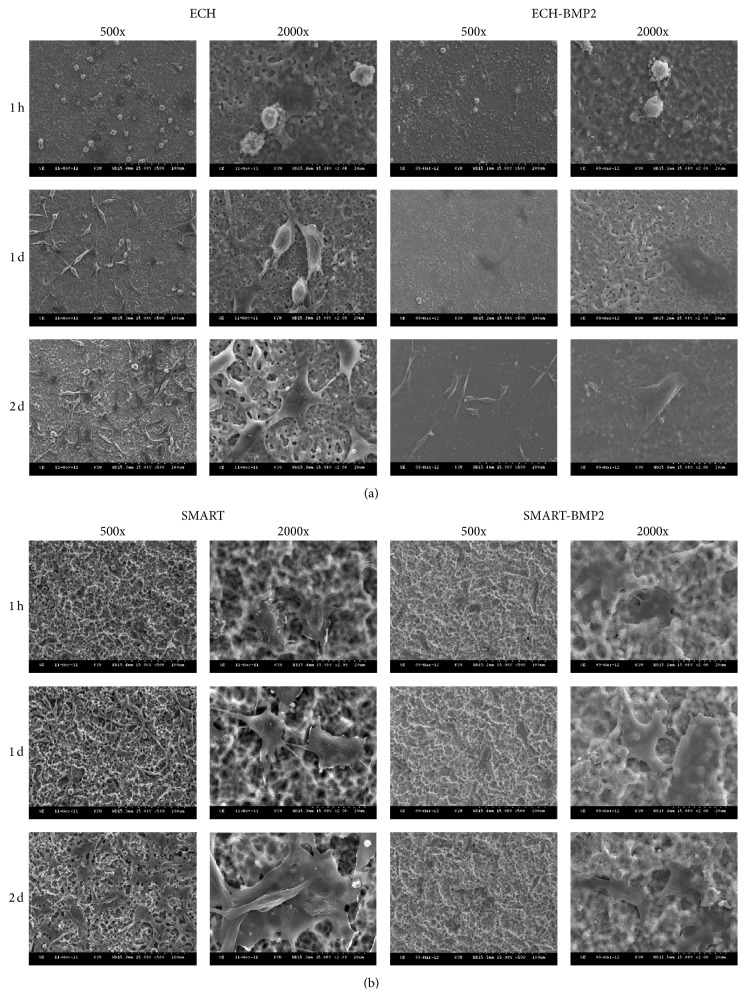
Cell morphology on various Ti surfaces. Micrograph images were observed using SEM of D1 cells on ECH (a) and SMART (b) surfaces coated with or without BMP-2 after 1 h, 1 d, and 2 d (ECH-B: ECH-BMP-2; SMART-B: SMART-BMP-2).

In evaluating the biocompatibility of implants, we observed cell attachment ability and proliferation. In this work, standard cell attachment was first established for cell culturing to determine the accurate biological performance in cell growth and activity in vitro.  Figure 2 displays the micrograph images of D1 cells on various Ti surfaces with or without BMP-2 after 1 h, 1 d, and 2 d. After 1 h of incubation, the cells on the specimen without BMP-2 coating were spherical, and numerous microvilli were found. After 1 d of culture, the cells attached more effectively to the surfaces, thereby supporting the trend observed after 1 h of culture. The SMART group without BMP-2 coating had more extended cell morphology than that of the ECH group without BMP-2 after 1 d of cell culture. After 1 and 2 d of culture, the cells attached more effectively to the surfaces and cells showed similar morphologies whose all filopodia were extended outward compared with the 1 h cultures. The result showed that the BMP-2 coating surface with ECH and SMART enhanced cell attachment.

### 3.3. Cell Proliferation on Various Ti Implants

**Figure 3:**
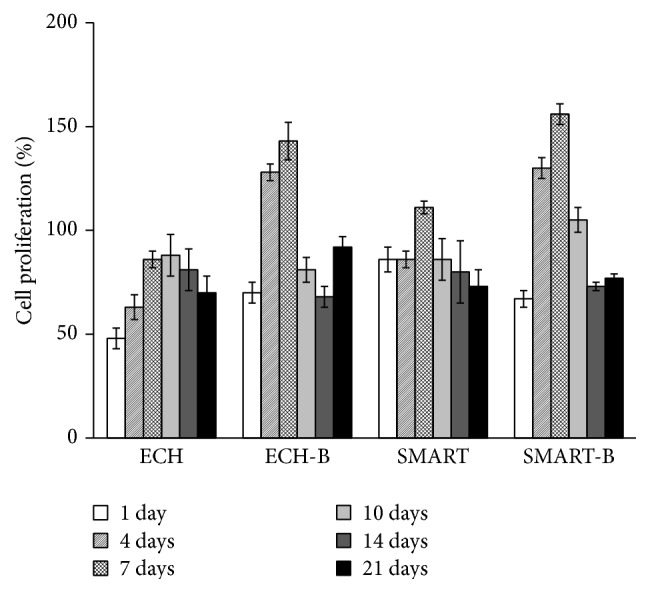
Cell proliferation on various Ti surfaces was detected using an MTS assay. Percentage of D1 cell proliferation on various Ti surfaces coated with or without BMP-2 for 1, 4, 7, 10, 14, and 21 d (*n* = 5) (ECH-B: ECH-BMP-2; SMART-B: SMART-BMP-2).

Cell proliferation was determined through MTS assay for 1, 4, 7, 10, 14, and 21 d. The D1 cell proliferation on the experimental groups compared with the machined groups is shown in Figure 3. The number of D1 cells increased on Day 1 until Day 7. All the metabolic activities reached the greatest value after Day 7 of D1 cell cultures in all groups. Furthermore, the D1 cells in the SMART-B group had the highest metabolic activity among these in all groups. The dramatic change of values, during which the number of cells decreased from Day 10 to Day 21, showed the mineralized mechanism occurred particularly on the surfaces of the ECH-B and SMART-B groups.

### 3.4. ALP Activity of D1 Cells Seeded on Various Ti Implants

**Figure 4:**
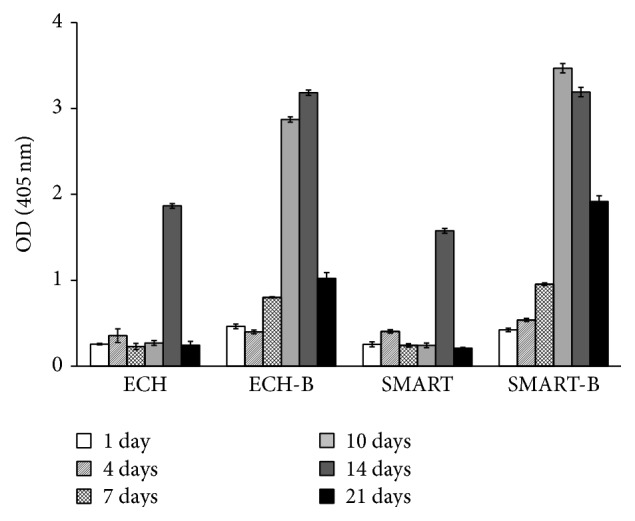
ALP activity of D1 cells seeded on various Ti surfaces. ALP activity of D1 cells on various Ti surfaces coated with or without BMP-2 for 4, 7, 10, 14, and 21 d (*n* = 5) (ECH-B: ECH-BMP-2; SMART-B: SMART-BMP-2).

The primary goal of this study was to differentiate D1 cells to osteoblast and mineralization. The ALP activity of D1 cells is displayed in Figure 4. The ALP activity of D1 cells was measured after 1, 4, 7, 10, 14, and 21 d of culture on the various treated Ti substrates. After incubation for 1, 4, and 7 d, the difference in ALP activity was not statistically significant for cells cultured on all surfaces. The ALP activity reached its maximum after 14 d for ECH and SMART, particularly in the BMP-2 coating groups. Notably, ALP activity shifted to an earlier time on Day 10 of D1 culture to reach its maximum, which occurred in the group with the BMP-2 bonding surface. Therefore, the combined factors of surface morphologies and BMP-2 growth led to earlier expressions of ALP on Ti substrate bonding with BMP-2.

### 3.5. Initial Bone Implant Contact of Various Ti Implants at 14 Days

**Figure 5:**
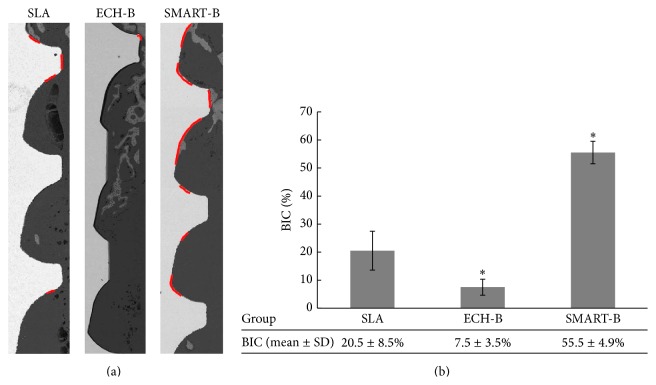
Initial BIC of various Ti implants was analyzed using SEM at 14 days. FE-SEM image (a) shows the BIC in SLA, ECH-B, and SMART-B groups. Statistical summary presenting the BIC estimated means ± standard error for the various implant surfaces (b) (ECH-B: ECH-BMP-2; SMART-B: SMART-BMP-2).

**Figure 6:**
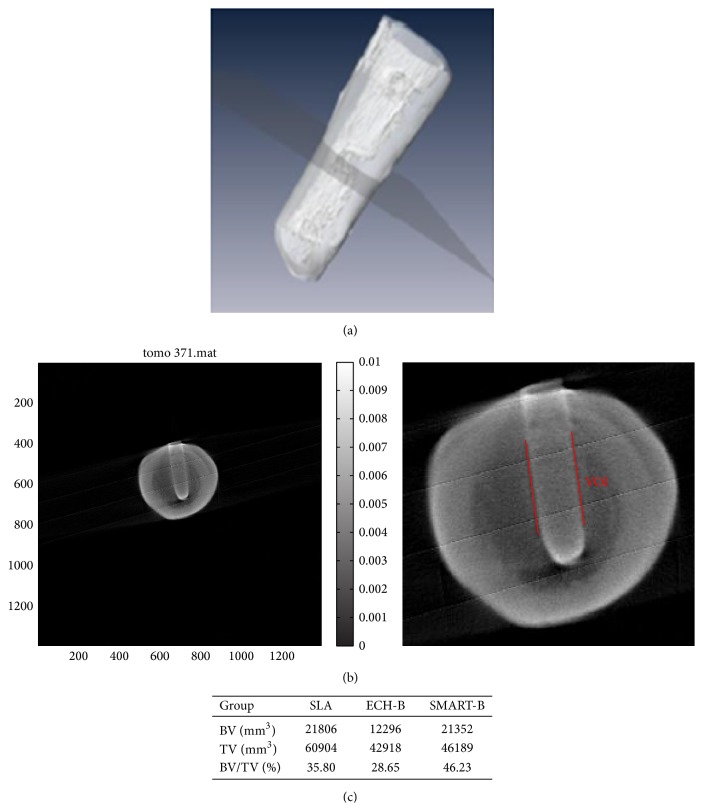
Bone quality evaluation at dental implant site by using micro-CT at 14 days. 3D image was observed using micro-CT (a) and the transverse section (b) was used to analyze the parameter of bone volume, total volume, and BV/TV (c) (ECH-B: ECH-BMP-2; SMART-B: SMART-BMP-2).

The most commonly used commercialized method of modifying implant surfaces of a SLA group was selected for comparison with our experimental groups of ECH-B, SMART, and SMART-B in our animal study. Bone implant contact (BIC) was measured through micro-CT. FE-SEM imaging precisely showed the images of the bone implant contact at 14 days (Figure 5(a)), leading to the BIC quantifications (Figure 5(b)). The highest value was presented in the SMART-BMP-2 bonding group. However, the ECH-BMP-2 group showed lower BIC than the SLA groups. The micro-CT analysis showed the same BIC tendency in the SMART-BMP-2 group, in which the BV/TV was significantly higher than in the SLA and ECH-BMP-2 groups (Figures 6(a), 6(b), and 6(c)).

## 4. Discussion

The silanization modified surfaces, which we used successfully in previous studies [6, 13], allowed bioactive functional groups for increased adhesion abilities and biological properties. The homogenous distribution of BMP-2 among the surfaces is shown in Figure 1(a). The declined contact angle indicated the enhanced wettability after the BMP-2 bonding with the functional substrates. The dramatic change in the M after the self-assembled surface of silane bonding with BMP-2 showed the modified wettability.

The modified surfaces with bioactive proteins, BMP-2, immediately provided osteogenic signals, enhancing the cell into a mechanical three-dimensional structure and thus attaching onto surfaces because of the anchored and covalently bonding BMP-2 in the pores. Although the results showed similar patterns after 2 d of D1 progenitor cells in terms of morphological characteristics, the thin film bonding over the surface stimulated higher cell proliferation and mineralization after 7 d, as shown in Figures 3 and 4. As previously mentioned, the clinical challenges in delivering BMP-2 in implant sites are the possibility of denaturation or deactivation of proteins. These results demonstrate that BMP-2 can potentially apply in bone regeneration and reduce healing time. Furthermore, this modified surface structure for cell anchorage and extension plays a more critical role than delivery in the soluble BMP-2. Crouzier et al. demonstrated that a high concentration of ligand and cytoskeleton remodeling was induced by matrix-bound BMP-2 but could not be observed in diffuse soluble BMP-2 [16]. This phenomenon was also shown in our previous study [17].

To evaluate the initial degree of osseointegration of implants, the ECH-B and SMART-Bwith the commercialized SLA treatment were compared in the animal studies. The results of the SMART-B group showed approximately 50% BIC and BV/TV at as early as 14 d. Notably, although the ECH-B group demonstrated effective cell proliferation and mineralization in vitro, the lowest value of BIC was observed in the ECH-B groups. The inconsistent results probably occurred as a result of losing physical anchorage because the roughness of ECH-B (mean 0.32 *μ*m) was significantly less than SMART-B (1.27 *μ*m), which led to the loss of the bonding of BMP-2 during surgical procedures for ECH-B implant placement. The higher roughness with chemical bonding in the SMART-B group exhibited superior resistance to friction between the bone and modified surfaces. Accordingly, the average roughness of the ECH-B group was flatter than the SMART-B group. The surface roughness may be the reason leading to the loss of the bonding of BMP-2. The apparent gap area of the SMART-B group provides evidence of close bonding to the BMP-2 by silanization, as indicated by the red line in Figure 5.

## 5. Conclusions

The interaction between osteoprogenitor cells and BMP-2 modified surfaces for Ti dental implants can be increased to enhance cell mineralization. Through silanization, the BMP-2 protein can improve proliferation and enhance ALP expression at the early stages of cell culture, which was particularly expressed in the SMART group in our study. The results suggest that an active biological function surface can be designed to accelerate mineralization of osteoprogenitor cells, but this should be tested in animal studies to confirm the results. The surfaces are suitable for all properties, such as roughness, wettability, and biological factors, which can particularly affect the early osseointegration of dental implants. Therefore, further studies should focus on the degrees of osseointegration of dental implants for various pretreatment implant surfaces before silanization can influence this process at the early stages.
